# Current Choices and Management of Treatment in Persons with Severe Hemophilia A without Inhibitors: A Mini-Delphi Consensus

**DOI:** 10.3390/jcm11030801

**Published:** 2022-02-02

**Authors:** Antonio Coppola, Massimo Franchini, Giovanni Pappagallo, Alessandra Borchiellini, Raimondo De Cristofaro, Angelo Claudio Molinari, Rita Carlotta Santoro, Cristina Santoro, Annarita Tagliaferri

**Affiliations:** 1 Regional Reference Centre for Inherited Bleeding Disorders, University Hospital of Parma, 43121 Parma, Italy; atagliaferri@ao.pr.it; 2 Department of Hematology and Transfusion Medicine, Carlo Poma Hospital, 46100 Mantova, Italy; massimo.franchini@asst-mantova.it; 3 School of Clinical Methodology, IRCCS “Sacro Cuore-Don Calabria” Hospital, 37024 Negrar di Valpolicella, Italy; giovanni.pappagallo@me.com; 4 Regional Reference Centre for Adult Hemorrhagic and Thrombotic Disorders, Hematology, City of Health and Science University Hospital, 10126 Turin, Italy; alesandre@libero.it; 5 Centre for Hemorrhagic and Thrombotic Diseases, IRCCS A. Gemelli University Hospital Foundation, 00168 Rome, Italy; raimondo.decristofaro@unicatt.it; 6 Department of Translational Medicine and Surgery, Sacro Cuore Catholic University, 00168 Rome, Italy; 7 Regional Reference Centre for Hemorrhagic Diseases, Thrombosis and Hemostasis Unit, IRCCS Giannina Gaslini Hospital, 16147 Genova, Italy; aclaudiomolinari@gaslini.org; 8 Centre for Hemorrhagic and Thrombotic Disorders, Pugliese Ciaccio Hospital, 88100 Catanzaro, Italy; ritacarlottasantoro@gmail.com; 9 Hematology, University Hospital Policlinico Umberto I, 00161 Rome, Italy; santoro@bce.uniroma1.it

**Keywords:** hemophilia A, factor VIII concentrates, extended-half-life factor VIII concentrates, emicizumab, non-replacement therapy, prophylaxis, mini-Delphi, consensus

## Abstract

Background. Regular treatment to prevent bleeding and consequent joint deterioration (prophylaxis) is the standard of care for persons with severe hemophilia A, traditionally based on intravenous infusions of the deficient clotting FVIII concentrates (CFCs). In recent years, extended half-life (EHL) CFCs and the non-replacement agent emicizumab, subcutaneously administered, have reduced the treatment burden. Methods. To compare and integrate the opinions on the different therapies available, eight hemophilia specialists were involved in drafting items of interest and relative statements through the Estimate-Talk-Estimate (ETE) method (“mini-Delphi”), in this way reaching consensus. Results. Eighteen items were identified, then harmonized to 10, and a statement was generated for each. These statements highlight the importance of personalized prophylaxis regimens. CFCs, particularly EHL products, seem more suitable for this, despite the challenging intravenous (i.v.) administration. Limited real-world experience, particularly in some clinical settings, and the lack of evidence on long-term safety and efficacy of non-replacement agents, require careful individual risk/benefit assessment and multidisciplinary data collection. Conclusions. The increased treatment options extend the opportunities of personalized prophylaxis, the mainstay of modern management of hemophilia. Close, long-term clinical and laboratory follow-up of patients using newer therapeutic approaches by specialized hemophilia treatment centers is needed.

## 1. Introduction

Hemophilia A is a rare X-linked coagulation disorder (1:5000 male live births) caused by gene variants affecting the synthesis or function of factor VIII (FVIII), an essential component of the intrinsic pathway of blood coagulation [1,2]. Residual FVIII plasma levels define severe, moderate or mild hemophilia (<1%, 1–5% and >5–40%, respectively), which substantially correlates with the severity of bleeding tendency [2,3]. 

The treatment of hemophilia A is traditionally based on intravenous (i.v.) infusion of the deficient clotting FVIII concentrates (CFCs; replacement therapy), usually performed by the patients themselves or their caregivers in a home treatment setting [4], when bleeding episodes occur (on demand) or to prevent them (prophylaxis). Long-term, regular prophylaxis with FVIII concentrates, aimed at preventing in particular bleeding into joints to avoid or delay the development of hemophilic arthropathy, thus enabling an active lifestyle and satisfactory quality of life, has been recognized since the 1990s as the standard of care in patients with severe hemophilia A [5,6,7] and, more recently, even in those with moderate forms with relevant bleeding manifestations [4,8]. Over more than half a century, clinical studies, including randomized trials, documented the benefits of prophylaxis in preventing bleeding and joint morbidity when started early in children, after no more than one major joint bleed and before 3 years of age (primary prophylaxis), or after few bleedings (early secondary prophylaxis), in the absence of signs of joint damage [8,9,10,11]. However, clear advantages in reducing bleeding and its deleterious effects on joint status and quality of life have been shown even in patients starting prophylaxis later in life, in adolescence and adulthood [8,12,13]. However, the main downside is the need for high-frequency i.v. infusions (usually three times per week), which lead to difficult implementation in children and patients with poor venous access and, overall, adherence problems [14,15,16,17]. This issue may be partially overcome with the use of extended half-life (EHL) concentrates, which increase factor trough levels and permit less frequent—although still i.v.—dosing, thus reducing prophylaxis burden and improving treatment adherence and personalization [18,19]. Different approaches based on alternative hemostatic agents to substitute for CFC have arisen in the last few years [20]. These innovative products (i.e., non-factor replacement therapies) act by mimicking FVIII in tenase complex formation, or inhibiting naturally occurring anticoagulant proteins or inhibitors of activation of coagulation, thus enhancing thrombin generation and rendering fibrin clots more resistant; importantly, their prolonged half-life and subcutaneous (s.c.) administration route may play a crucial role for prophylaxis implementation and adherence, especially for patients with poor venous access [20,21]. To date, emicizumab, a bispecific humanized monoclonal antibody mimetic of FVIII and able to promote the activation of Factor X [22], is the only approved non-replacement treatment for hemophilia A [23,24]. Prophylaxis with emicizumab has been shown to be safe and able to prevent bleeds in patients with hemophilia A with and without inhibitors [24,25,26,27]. Clinical experience is still limited in such a novel treatment, and data on its long-term safety and efficacy are lacking [8,28,29]. 

The presence of well-established (CFCs) and novel and effective (EHL CFCs and non-factor replacement therapies) products pose a great number of questions on which are the best options and how to handle them in patients with different clinical features and aims of treatment (according to age, lifestyle and physical activity, joint status, presence of cardiovascular (CV) or other comorbidities). Moreover, specific issues should be considered, including the management of intercurrent bleeding and invasive procedures, product switch and the utility of pharmacokinetics studies, laboratory monitoring, and long-term outcomes. In the frame of this evolving scenario, the present study aims to explore, compare and integrate the opinions of Italian hemophilia specialists on (1) the different therapies available today; (2) their current implementation in clinical practice in various settings, from routine prophylaxis to emergency and surgery; and (3) relevant issues concerning management and outcomes of patients on these different products.

## 2. Methods

**Figure 1:**
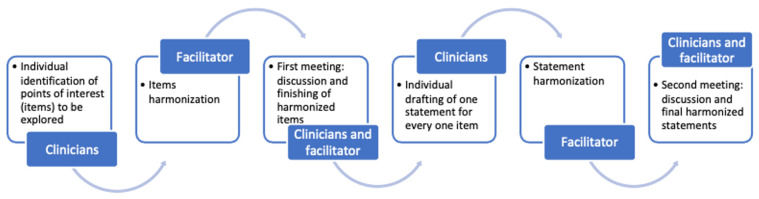
Project flowchart.

The flowchart (Figure 1) offers an overview of the project workflow, with the use of the Estimate-Talk-Estimate (ETE) method, or “mini-Delphi” [30,31].

The ETE is a method for reaching consensus within a selected group of experts. It combines a nominal group activity restricting verbal interaction with face-to-face interaction processes. Firstly, experts individually generated some points of interest (items) which, in their opinion, deserved to be explored and discussed. A senior clinical epidemiologist (GP) expert in gaining consensus among stakeholders (facilitator) harmonized these items, which were then presented to the expert group. During the first meeting, harmonized items were discussed face-to-face to reach an agreement between the facilitator’s work and the experts’ opinions. Afterward, finalized items were used by clinicians to draft one statement for every one item individually. This process resulted in a certain number of statements, which were then harmonized by the facilitator. In the second and last face-to-face meeting, the experts and the facilitator reviewed and further discussed the harmonized statements, reaching a final version. Statements generated in this way expressed consensus among the experts involved.

The expert panel comprised eight clinicians involved in the global care of hemophilia at different Italian centers, with heterogeneous settings (only pediatric, mainly adults, both pediatric and adult patients), healthcare organization (inpatient/outpatient, on-call and emergency availability, general or specialized laboratories) and background expertise—namely general and clinic hematology, internal medicine, pediatrics, oncology, and endocrinology—in order to achieve a broader overview of the issues. Due to the nature of the consensus technique, the panel sought the assistance of a facilitator to provide material preparation, meeting facilitation, and scientific and methodological accuracy.

## 3. Results

According to the previously mentioned process, the eight hemophilia experts identified 18 points of interest regarding the treatment of severe hemophilia A patients without inhibitors. These points were then harmonized through the assistance of the facilitator with the generation of 10 final items. For each item, after the harmonization of individual drafts, a statement was generated during a plenary session with the presence of all the members of the expert panel. Table 1 shows the harmonized items with the final generated statements.

## 4. Discussion

This consensus work reports the conclusions of an expert panel composed of eight clinicians with various expertise, coming from different settings and healthcare organizations, focusing on treating severe hemophilia A patients without inhibitors. Addressing this issue in such a fashion is of utmost importance nowadays, as the optimal care in this setting requires comprehensive approaches provided by a multidisciplinary team of specialists [8,32,33].

Prophylaxis (whether primary, secondary or tertiary) is currently the universally recognized treatment of choice (statement 1) [4,8,34,35,36]; however, most available data arise from studies with SHL FVIII CFCs [8,9,10,11,12,13,37]; therefore, the rise of novel prophylaxis regimens with EHL FVIII CFCs or non-replacement products requires careful and thoughtful assessments due to a lack of clear guidelines and evidence, especially long-term [18,19,38,39,40]. In this rapidly changing therapeutic landscape, the ETE method was applied to some topical and still controversial issues. This approach was chosen because of its superiority in giving correct estimates in judgmental tasks [30]. Indeed, the experts sorted out their thoughts based on the literature and their clinical experience, avoiding the influence of intragroup and socio-emotional dynamics. The drafted items and statements represented a starting point for identifying contents of greater shared interest in this area. 

In agreement with the recently updated World Federation of Hemophilia (WFH) recommendations, the main topic of interest is the importance of tailored treatment, able to consider the overall individual clinical needs (bleeding phenotype, joint status, lifestyle and physical activity) and to ensure patients the best possible quality of life, substantially comparable to non-coagulopathic population [8]. Indeed, despite these statements focusing on different topics, virtually all converge on the importance of a personalized approach to treatment. This is clearly shown in statements 1 (“The type and regimen of treatment should be tailored to the patient′s needs and lifestyle”), 4 (“personalized regimens”), 7 (“prophylaxis personalization”), 8 (“customization of the prophylaxis regimen”), and 10 (“The evaluation of the individual pharmacokinetic response […] is essential for the personalization of the prophylaxis regimen”). While not explicitly stated, other statements address the issue of a tailored path of care, in the case of management problems (statements 3 and 9), lack of strong evidence regarding novel treatments (statements 4 and 6), and bleeding or risk of bleeding (statement 5). The availability of multiple products with different characteristics is helpful to individualize treatment choices. This approach should be applied in all patients [8,34,35,36], particularly in those with additional needs. In this respect, highlighting the importance of an early start of primary prophylaxis, the panel recognized the facilitation provided by the subcutaneous administration of non-replacement treatment, i.e., emicizumab, to date the only licensed product (statement 3). This choice can be crucial in all patients with venous access problems, particularly very young children, in whom effective prophylaxis could be started even earlier than usually done [8,38], thus enabling protection from severe unless rare bleeding [41]. Another specific need regards the growing number of hemophilic patients, in parallel with their increased life expectancy [42], with thrombotic/cardiovascular risk/diseases requiring antithrombotic treatments. In these cases, physicians should carefully adjust prophylaxis regimens to the enhanced bleeding risk due to antiplatelet or anticoagulant drugs [43,44], on the other hand considering the underlying cardiovascular risk, given the morbidity and mortality substantially comparable to those of the general population and an increased prevalence of some risk factors, including hypertension [45,46]. In such cases, as well in other patients with high protection needs, for example, due to intense physical activities [47], the choice for FVIII CFCs enables extensive individualization of prophylaxis based on doses and, in particular, frequency of infusions vs. the relatively fixed although sustained protection from non-replacement treatment. 

According to the expert panel, the availability of EHL products further increased personalized treatment opportunities. Indeed, while showing the same efficacy in preventing and treating bleeds, EHL products allow individualized prophylaxis regimens by reducing infusion frequency, thus improving patients’ satisfaction and adherence to treatment, or increasing protection and FVIII trough levels by maintaining more frequent administration (statement 8) [18,19,48,49]. Although the reduction of infusions and treatment burden is the most important need for patients and caregivers [50], higher protection, thanks to more sustained FVIII through levels, should be addressed not only in patients with higher bleeding risks but particularly in light of data showing the development of arthropathy even in patients on well-conducted prophylaxis with standard products and target levels [17]. When switching from an SHL to an EHL product, patients need to be trained to adjust treatment management and lifestyle to the new regimen and identify early the possible changes in efficacy and safety. Therefore, patients and their caregivers should be prepared for a phase of intensive clinical/laboratory monitoring, which is crucial to personalize and optimize regimens and outcomes of prophylaxis [48,51]. An assessment of the individual pharmacokinetic (PK) response should precede the switch (statement 10) [18], revealing the advantages of the new product and providing information for individualized regimens. Many studies have shown the utility of PK in hemophilia treatment due to the wide inter-individual variability in CFC PK; indeed, this approach is associated with better outcomes compared to standard prophylaxis, even from the pharmacoeconomic perspective [52,53,54,55].

In patients on prophylaxis with emicizumab, in the case of intercurrent bleeding events and minor or major invasive procedures, both standard (SHL) and EHL FVIII concentrates can be used for the mandatory adjunctive treatment. Although real-world evidence is being published [56,57], clinical experience in this setting is still limited; therefore, the direct management or at least supervision of the hemophilia treatment centers (HTCs) is advised (statement 5) [58]. In this regard, the monitoring of FVIII levels should be available using the chromogenic assay, in this case with bovine reagents, insensible to emicizumab (statement 7) [59]. Overall, the chromogenic method is increasingly considered the preferred assay for FVIII measurements, considering the discrepancies shown by one-stage assays, particularly in patients on treatment with modified FVIII concentrates, including EHL products [60,61,62]. Laboratory monitoring accuracy is crucial for personalizing prophylaxis regimens and optimizing treatment outcomes; in this respect, a continuous interaction between clinicians and the laboratory team is needed.

The absence of long-term evidence is a major challenge in assessing novel hemophilia treatments [8,20,58]. Therefore, if EHL or non-replacement products are used for prophylaxis, patients should undergo careful clinical and laboratory follow-up, possibly overseen by experienced HTCs. Long-term data collection is relevant for safety issues, particularly for pegylated products [63] and non-replacement therapy (statement 6) [28,29]. Moreover, achieving evidence about the prevention of joint damage by non-replacement treatment is highly needed to fully support the efficacy of prophylaxis (statement 3) [8,58]. With this aim, monitoring joint health in patients on emicizumab prophylaxis is an essential task [38]. The expert panel outlines the need for appropriate clinometric tools to be used on a regular basis. Among clinical measurements, the Hemophilia Joint Health Score (HJHS) is the most extensively studied, providing data about joint structure and function [64]. This information should be integrated with imaging to prevent and detect arthropathy early. With this aim, scores based on the easily available and repeatable joint ultrasound are increasingly adopted. Experienced personnel should perform these multidisciplinary assessments to optimize reliability and standardize measurement procedures [65]. Overall, clinicians should weigh these unanswered issues against the excellent results in preventing bleeding and the undoubted advantages of the easier s.c. administration and prolonged half-life with reduced dosing frequency, facilitating implementation and adherence to treatment [8,28,29]. Individualized choices, taking into consideration not only clinical issues but also patients’ self-assessment and preferences, are recommended by the recent WFH guidelines, which update the definition of prophylaxis considering all therapeutic products regularly administered to maintain hemostasis to prevent bleeding, joint deterioration and impairment of quality of life. This includes emicizumab, for which further research is needed, and other non-replacement agents, whose data are presently much more limited [8]. 

This study has some limitations. Being a consensus work, it cannot produce empirical data. Moreover, it is limited to Italian settings. Thus, statements could not align with other countries’ scenarios, which can differ because of different regulations and resources. Eventually, the process of statement drafting may result in statements being redundant or clearly addressed in the literature.

## 5. Conclusions

Several newer (replacement and non-replacement) treatment products for hemophilia A characterized by an extended half-life have been available in the last few years. These innovative agents are potentially attractive, offering the advantage of reducing the frequency of dosing and improving patients′ adherence to therapy. The increased treatment options extend the opportunities of individualization of prophylaxis, which is the mainstay of modern management of hemophilia. Treatment choices in the individual patient can currently consider clinical needs in terms of protection from bleeding and even specific issues to facilitate treatment and adherence. In this perspective, CFCs, particularly EHL products, seem more suitable for personalized prophylaxis regimens, although the i.v. administration remains challenging in some patients, particularly young children. Regarding non-replacement treatment, limited experience in some clinical settings and the lack of evidence on long-term safety and impact in preserving joint status require careful individual assessment of the possible advantages and disadvantages. Overall, a close clinical and laboratory follow-up and multidisciplinary data collection during the use of all newer therapeutic approaches are required to be performed at specialized HTCs with appropriate tools and assays in the long term.

## Figures and Tables

**Table 1:** Final items and statements.

	Final Items	Final Statements
1	Implementation, personalization, and adherence of prophylaxis in persons with severe (and moderate) hemophilia	Regular long-term prophylaxis to prevent bleeding, preserve joint status, and ensure a quality of life equal to peers without hemophilia is the treatment of choice in patients of all ages with severe hemophilia A or severe hemorrhagic phenotype. The type and regimen of treatment should be tailored to the patient′s needs and lifestyle.
2	Early prophylaxis in children	It is essential to start primary prophylaxis early to prevent serious bleeding and the onset of joint damage. Although there is a lack of long-term evidence, the non-replacement product s.c. administered can be used in case of poor venous access and/or other serious problems affecting feasibility of prophylaxis with intravenous infusions of CFCs.
3	Prevention of arthropathy in patients receiving non-replacement therapies	If prophylaxis with non-replacement therapy is chosen, in the absence of long-term evidence demonstrating its efficacy in preventing hemophilic arthropathy, standardized prospective monitoring of the joint condition with suitable clinometric (clinical and imaging) tools is appropriate.
4	Prophylaxis in patients with concomitant cardiovascular risk and antithrombotic therapy	In the presence of cardiovascular risk factors and/or comorbidities and indications for antithrombotic therapy, it is necessary to establish prophylaxis with personalized regimens of FVIII concentrates that balance hemorrhagic and thrombotic risk. There is currently no evidence about the safe, optimal level of VIII in different clinical situations.
5	Management of intercurrent bleeding and invasive procedures during non-replacement therapy	Therapy with FVIII is mandatory in cases of intercurrent bleeding, invasive procedures at high hemorrhagic risk, and/or major surgery in patients on prophylaxis with non-replacement therapy. Bleeding episodes and maneuvers at risk should be managed by hemophilia centers, where appropriate clinical and laboratory assessments are available.
6	Long-term safety (replacement and non-replacement therapies)	Long-term data support the safety of standard half-life FVIII concentrates, while there is still no direct evidence regarding extended half-life concentrates, particularly for pegylated products and non-replacement therapy. An adequate clinical and laboratory follow-up is therefore considered appropriate.
7	Laboratory monitoring (chromogenic assay, one-stage assay)	Monitoring of FVIII levels with appropriate tests is essential for prophylaxis personalization with CFCs and in the case of invasive procedures and surgery management. Considering the heterogeneity of the FVIII CFCs available and the discrepant results with the different “one-stage” reagents, as well as the interferences of emicizumab, the chromogenic method should be considered the assay of choice for the measurement of FVIII. This particularly applies to cases of treatment with pegylated CFCs and emicizumab. In the latter, bovine reagents should be used.
8	Product switch (standard half-life to extended half-life) and role of age, duration of the interval between infusions and safety	Switching from a standard half-life FVIII concentrate to an extended half-life product can allow adequate customization of the prophylaxis regimen, balancing the patient′s protection needs with convenience and adherence to treatment, thanks to the possible prolongation of the interval between infusions, according to the individual pharmacokinetic response.
9	Criteria for the use of non-replacement products	Prophylaxis with non-replacement products should be considered in patients with severe hemophilia A with difficulties in implementing and managing i.v. treatment with FVIII concentrates due to venous access problems, reduced adherence or other situations that hinder the regularity of therapy.
10	Utility of the pharmacokinetic study	Evaluating the individual pharmacokinetic response to an FVIII concentrate is essential for personalizing prophylaxis in relation to the specific therapeutic objectives, particularly when deciding and implementing a product switch and optimizing regimens, considering both the protection efficacy and convenience of treatment.

Abbreviations—CFC: clotting factor concentrate; FVIII: factor VIII; i.v. intravenous.

## Data Availability

No additional data outside of data presented in this manuscript are available.
